# Mesenchymal stem cells promote spermatogonial stem/progenitor cell pool and spermatogenesis in neonatal mice in vitro

**DOI:** 10.1038/s41598-022-15358-5

**Published:** 2022-07-07

**Authors:** Selin Önen, Sevil Köse, Nilgün Yersal, Petek Korkusuz

**Affiliations:** 1 Department of Stem Cell Sciences, Graduate School of Health Sciences, Hacettepe University, 06100 Ankara, Turkey; 2 Department of Medical Biology, Faculty of Medicine, Atilim University, 06830 Ankara, Turkey; 3 Department of Nutrition and Dietetics, Faculty of Health Sciences, Atilim University, 06830 Ankara, Turkey; 4 Department of Histology and Embryology, Faculty of Medicine, Gaziosmanpaşa University, 60030 Tokat, Turkey; 5 Department of Histology and Embryology, Faculty of Medicine, Hacettepe University, 06100 Sihhiye, Ankara, Turkey

**Keywords:** Infertility, Mesenchymal stem cells

## Abstract

Prepubertal cancer treatment leads to irreversible infertility in half of the male patients. Current in vitro spermatogenesis protocols and cryopreservation techniques are inadequate to expand spermatogonial stem/progenitor cells (SSPC) from testicles. Bone marrow derived mesenchymal stem cells (BM-MSC) bearing a close resemblance to Sertoli cells, improved spermatogenesis in animal models. We asked if a co-culture setup supported by syngeneic BM-MSC that contributes to the air–liquid interphase (ALI) could lead to survival, expansion and differentiation of SSPCs in vitro. We generated an ALI platform able to provide a real-time cellular paracrine contribution consisting of syngeneic BM-MSCs to neonatal C57BL/6 mice testes. We aimed to evaluate the efficacy of this culture system on SSPC pool expansion and spermatogenesis throughout a complete spermatogenic cycle by measuring the number of total germ cells (GC), the undifferentiated and differentiating spermatogonia, the spermatocytes and the spermatids. Furthermore, we evaluated the testicular cell cycle phases, the tubular and luminal areas using histochemical, immunohistochemical and flow cytometric techniques. Cultures in present of BM-MSCs displayed survival of ID4(+) spermatogonial stem cells (SSC), expansion of SALL4(+) and OCT4(+) SSPCs, VASA(+) total GCs and Ki67(+) proliferative cells at 42 days and an increased number of SCP3(+) spermatocytes and Acrosin(+) spermatids at 28 days. BM-MSCs increased the percentage of mitotic cells within the G2-M phase of the total testicular cell cycle increased for 7 days, preserved the cell viability for 42 days and induced testicular maturation by enlargement of the tubular and luminal area for 42 days in comparison to the control. The percentage of PLZF(+) SSPCs increased within the first 28 days of culture, after which the pool started to get smaller while the number of spermatocytes and spermatids increased simultaneously. Our findings established the efficacy of syngeneic BM-MSCs on the survival and expansion of the SSPC pool and differentiation of spermatogonia to round spermatids during in vitro culture of prepubertal mice testes for 42 days. This method may be helpful in providing alternative cures for male fertility by supporting in vitro differentiated spermatids that can be used for round spermatid injection (ROSI) to female oocyte in animal models. These findings can be further exploited for personalized cellular therapy strategies to cure male infertility of prepubertal cancer survivors in clinics.

## Introduction

Cancer treatment modalities used during childhood lead to infertility in 46% of male pediatric cancer survivors^1^. Spermatogonial stem cells remain the only existing option to preserve their fertility since spermatogenesis is not initiated in childhood yet. Sertoli cells reinforce self-renewal and differentiation of SSPCs by physicochemical interaction. The SSPCs constitute 0.3% and 22% of the male undifferentiated GC pool in rodents^2^ and humans^3^, respectively. The key regulatory factors of are ID4^4^, a selective marker of A_single_ spermatogonia, self-renewing SSCs and OCT4^5^, PLZF and SALL4^6^, common selective markers of A_single_, A_paired_, A_aligned_ spermatogonia, the SSPCs and play a crucial role in the survival and the self-renewal of SSC and SSPC pools. Cryopreservation methods are inadequate to protect the limited number of SSPCs in testicular sperm extraction (TESE) materials obtained before cancer treatment^7^. Current experimental procedures result in insufficient spermatogenesis after SSPC transplantation^8^.

Bone marrow derived mesenchymal stem and Sertoli cells have a similar embryonic origin, differentiation^9^ and immunomodulatory capacity^10^. Their proliferation and gene expression profile plays a critical role in spermatogenic regulation^11^. Direct injection of allogeneic BM-MSCs into testes increased the number of spermatogonia and repaired the testicular microenvironment in sterile rats^12–16^. BM-MSC injections suppressed the immune response responsible for the damage of GCs in busulfan treated mice^14,17,18^. Ex vivo preservation, long term culture and expansion of SSPCs is challenging because of low colony formation capacity in monolayer cell culture^19,20^. Although testis organ cultures represent a new platform in order to keep 3D niche conditions ex vivo, they are still inadequate in terms of providing sufficient number of GCs^21^. We hypothesized that an ALI co-culture system based on syngeneic BM-MSC could support survival, expansion and differentiation of the SSPC pool in vitro.

We therefore aimed to generate a novel ALI co-culture setup in which syngeneic BM-MSCs support Sertoli cells in long term culture of neonatal C57BL/6 mice testes. We evaluated the efficiency of this platform by quantifying tubular and luminal areas, the number of SSPCs, cell cycle phase of testicular cells, differentiating and total GCs on days 7, 14, 28 and 42 using flow cytometry (FCM), histomorphometry, and immunohistochemistry (IHC).

## Results

### Primary BM-MSCs are phenotypically and functionally characterized

**Figure 1:**
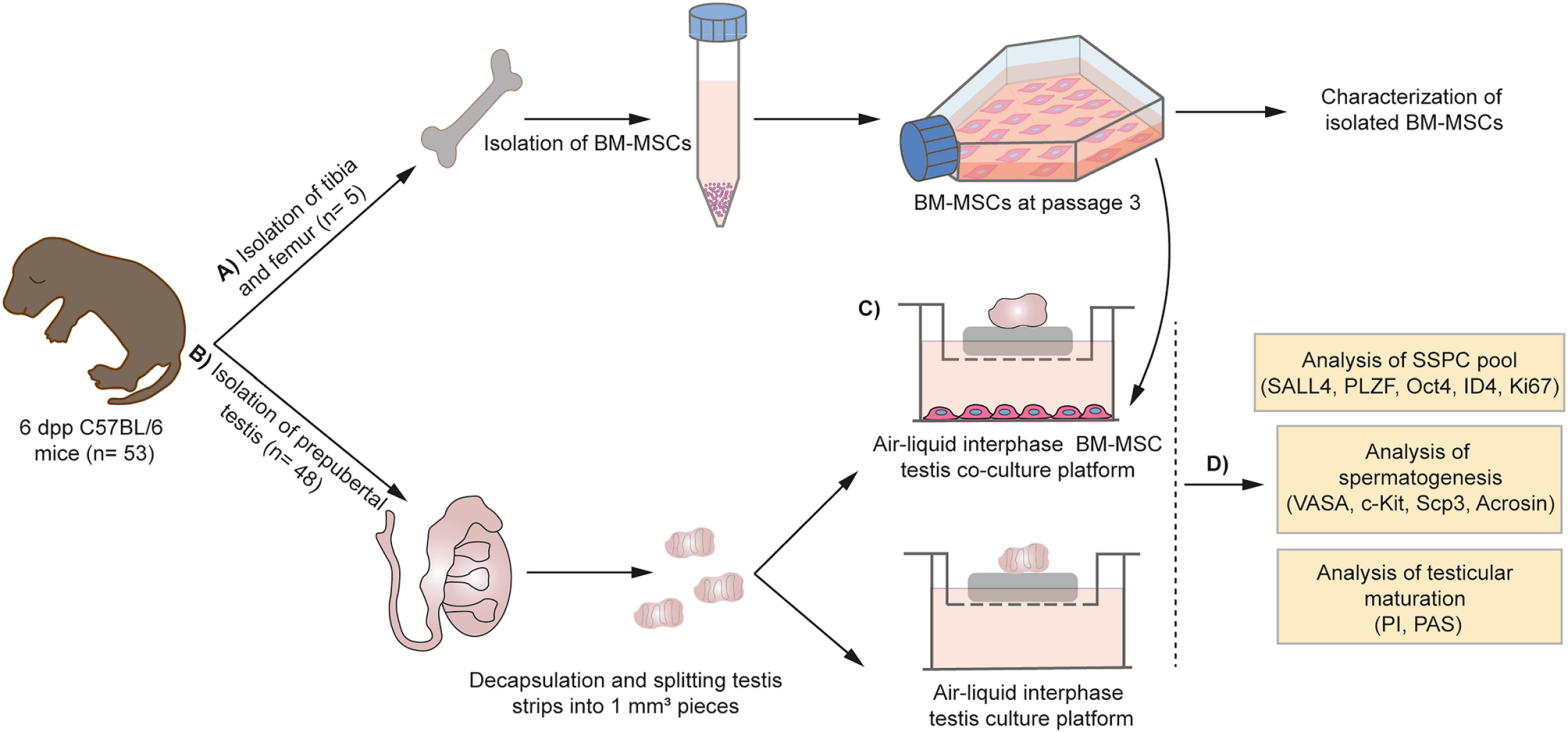
Schematic design of study. (**A**) The BM-MSCs were isolated from tibia and femur bones of 6-dpp neonatal mice (n = 5). Cells were characterized and used in further experiments at passage 3. (**B**) Neonatal testes were isolated, decapsulated, and seminiferous tubules were splitted into 1 mm^3^ pieces. (**C**) Seminiferous tubules were then taken onto agarose gels. And 0.4 µm pore sized transwell inserts were used to conduct the ALI culture platform. In BM-MSC co-culture group, BM-MSCs were used at the bottom of the 6-well culture plate. (**D**) When the 7, 14, 28 and 42-day culture periods have finished, testes were analyzed in terms of SSC, SSPC, differentiating GC and total GC number, cell cycle analysis, cell viability and testicular maturation by IHC, histology and FCM.

**Figure 2:**
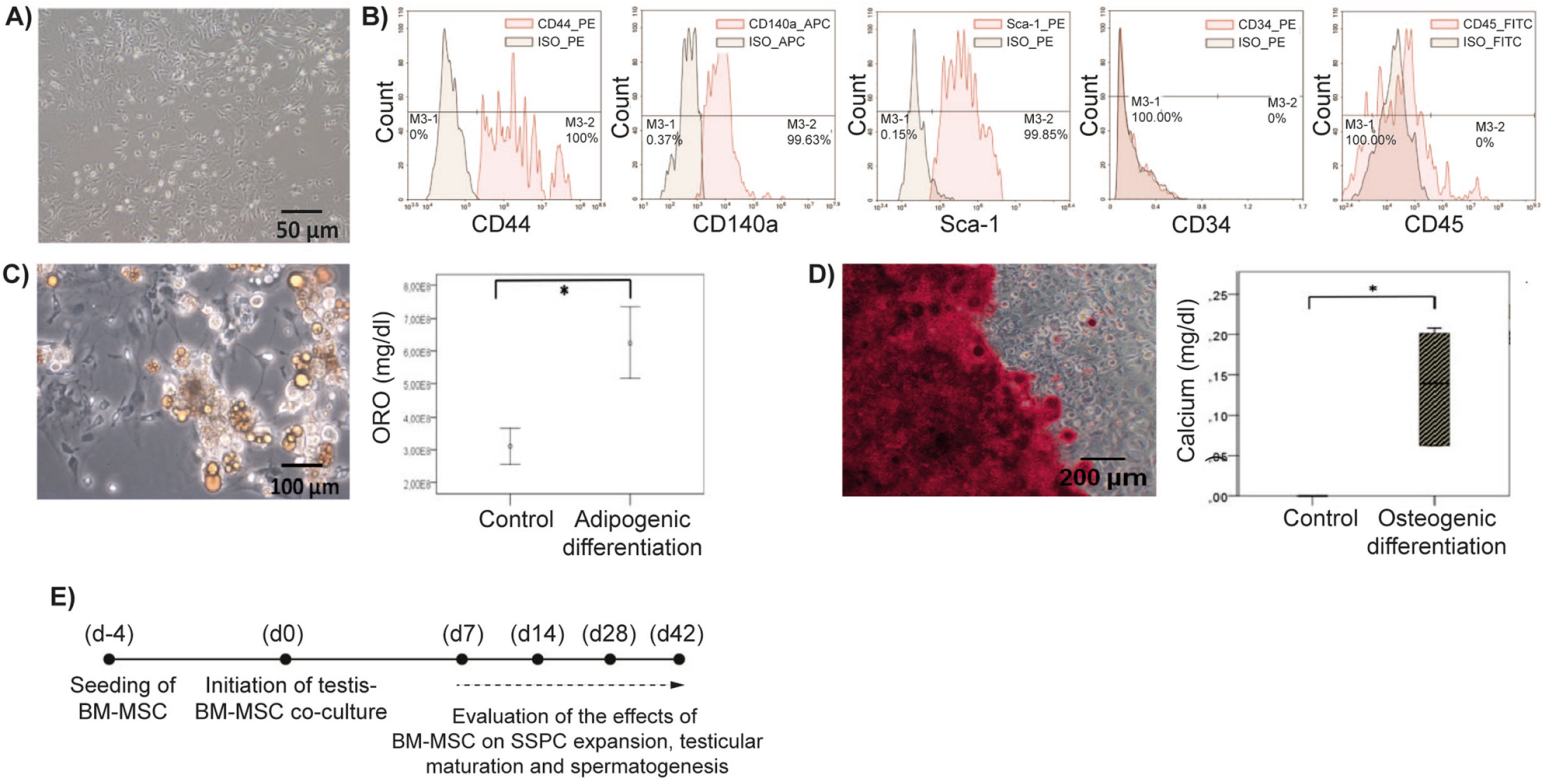
Characterization and workflow for BM-MSCs. (**A**) Micrograph of passage 3 spindle shaped BM-MSCs adhere to culture plate (100x). (**B**) FCM analysis of BM-MSCs expressing MSC surface markers (CD44, CD140a, Sca-1) positively and hematopoietic markers (CD34, CD45) negatively. BM-MSCs presents (**C**) adipogenic and (**D**) osteogenic differentiation (ORO, 200x; ARS, 100x, respectively, *p < 0.01). (**E**) Four day before the experiment, the MSCs were seeded into the wells and the insertion of testicular strips to the culture systems in both control and co-culture plates are accepted as day 0.

Experimental setup is illustrated schematically in Fig. 1. The BM-MSCs attached to the culture plates and presented a typical spindle-polygonal morphology at passage 3 (Fig. 2A). They homogenously (> 99%) expressed the mesenchymal stem cell (MSC) markers CD44, CD140a and Sca-1. Expression of the hematopoietic markers CD34 and CD45 was not detected (0%) (Fig. 2B). Cells differentiated into adipogenic (Fig. 2C) and osteogenic lineages (Fig. 2D) successfully. BM-MSCs were used in further steps of study in accordance with the experimental design (Fig. 2E).

### BM-MSCs induce the expansion of SSPCs in vitro

**Figure 3:**
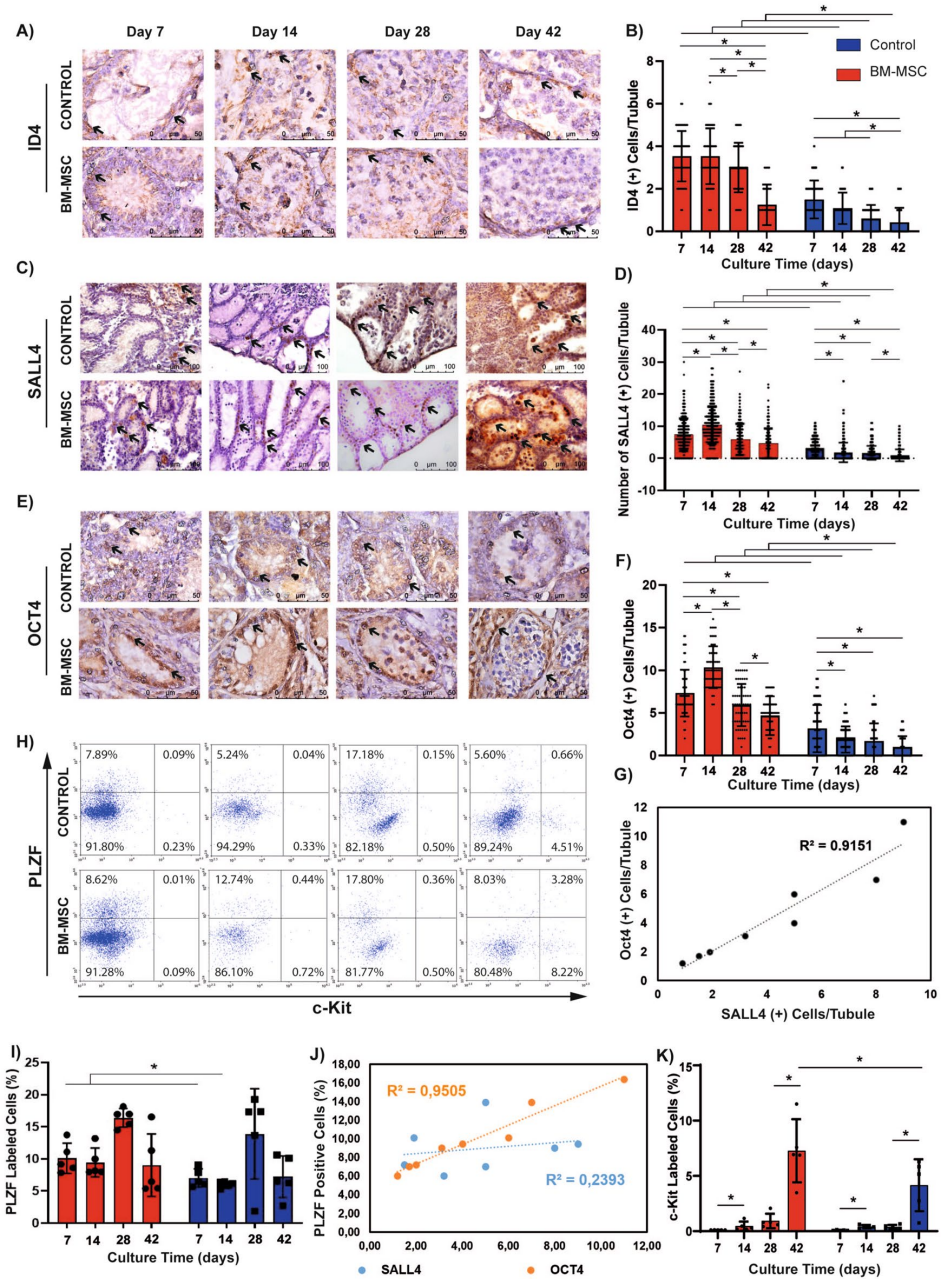
BM-MSCs induce the survival of ID4(+) SSCs, expansion of SALL4(+) and OCT4(+) SSPCs and c-Kit(+) differentiating GCs in vitro. Immune labeling of (**A**) ID4(+) SSCs, (**C**) SALL4 and (**E**) OCT4(+) SSPCs in neonatal mouse testes cultured for 7, 14, 28 and 42 days (ID4 and OCT4: 1000x, SALL4: 400x). The time dependent change in number of (**B**) ID4(+), (**D**) SALL4(+) and (**F**) OCT4(+) cells in BM-MSC co-culture and control group is illustrated in bar graph with standard deviation and data distribution (*p < 0.05, n = 6 testes, 300 tubules for SALL4, 60 tubules for ID4 and OCT4). (**G**) Line graph illustrates the positive correlation between OCT4 and SALL4 labelling for SSPCs in control and BM-MSC groups (R^2^ = 0.9151, p < 0.05). (**H**) Flow cytometry of PLZF and c-Kit in cultured testes. (**I**) Average PLZF expression in each group was shown in bar graph with standard deviation and distribution (*p < 0.05, n = 6). (**J**) Line graph illustrates the positive correlation of SALL4 and OCT4 with PLZF for SSPCs in control and BM-MSC groups (R^2^ = 0.9505 for OCT4, R^2^ = 0.2393 for SALL4, *p* < 0.05) (**K**) The expression of c-Kit in each group was shown in bar graph with standard deviation and distribution (*p < 0.05, n = 6).

BM-MSCs increased the number of ID4(+) SSCs per tubule between day 7 and 42 when compared to the controls by IHC for on days 7, 14, 28 and 42 (*p = *0.0001, Fig. 3A,B, Supplementary Fig. S1A). The BM-MSC co-culture group did not show a time dependent change between days 7 and 14 in ID4(+) cells (ns), but a decline in ID4(+) cells was observed from this point forward until day 42 (*p = *0.0001, Fig. 3B). A continuous decrease was detected in the number of ID4(+) cells from day 7 to 28 (*p = *0.0001) and day 14 to 42 (*p = *0.006) in the control.

BM-MSCs increased the number of both SALL4(+) (*p = *0.001, Fig. 3C,D, Supplementary Fig. S1B) and OCT4(+) (*p = *0.001, Fig. 3E,F, Supplementary Fig. S1C) SSPCs per tubule from day 7 to 42 when compared to the controls by IHC. The number of SALL4(+) (*p = *0.001) and OCT4(+) (*p = *0.001) cells increased from day 7 to 14 and exhibited a decline from this point forward until day 42 in the BM-MSC co-culture group (*p = *0.001, Fig. 3D,F). The control group presented a continuous decline in the number of SALL4(+) (*p = *0.001) and OCT4(+) (*p = *0.0001) cells from day 7 to 42. There was a strong positive correlation between OCT4 and SALL4 data on consecutive sections (R^2^ = 0.9151, *p = *0.0001, Fig. 3G) of IHC data of the BM-MSC co-culture and the control groups.

A significant increase was measured in the ratio of PLZF(+) SSPCs to total testicular cells in the BM-MSC co-culture group when compared to the control groups on days 7 and 14 by FCM (*p = *0.036, *p = *0.024, Fig. 3H,I). The PLZF(+) cell percentage increased slightly from day 14 to 28 and then declined from this point forward to day 42 in both groups (ns, Fig. 3I). The FCM data (PLZF) presented a positive correlation with the IHC data (SALL4 and OCT4) related to the number of SSPCs derived from in the control and the BM-MSC co-culture groups (R^2^ = 0.2393, *p = *0.024; R^2^ = 0.9505, *p = *0.0001 for SALL4 and OCT4, respectively, Fig. 3J).

### BM-MSCs promotes spermatogenesis in vitro

The proportion of c-Kit(+) differentiating spermatogenic cells to overall testicular cells was significantly higher in the BM-MSC co-culture group on day 42 (*p = *0.047, Fig. 3H,K) by FCM. It was similar in both the BM-MSC co-culture and control groups on days 7, 14 and 28 (Fig. 3K). The c-Kit(+) cell percentage from day 7 to 42; day 28 to 42 and day 14 to 42 time-dependently increased in both the BM-MSC co-culture and the control groups (*p = *0.001, Fig. 3K).

**Figure 4:**
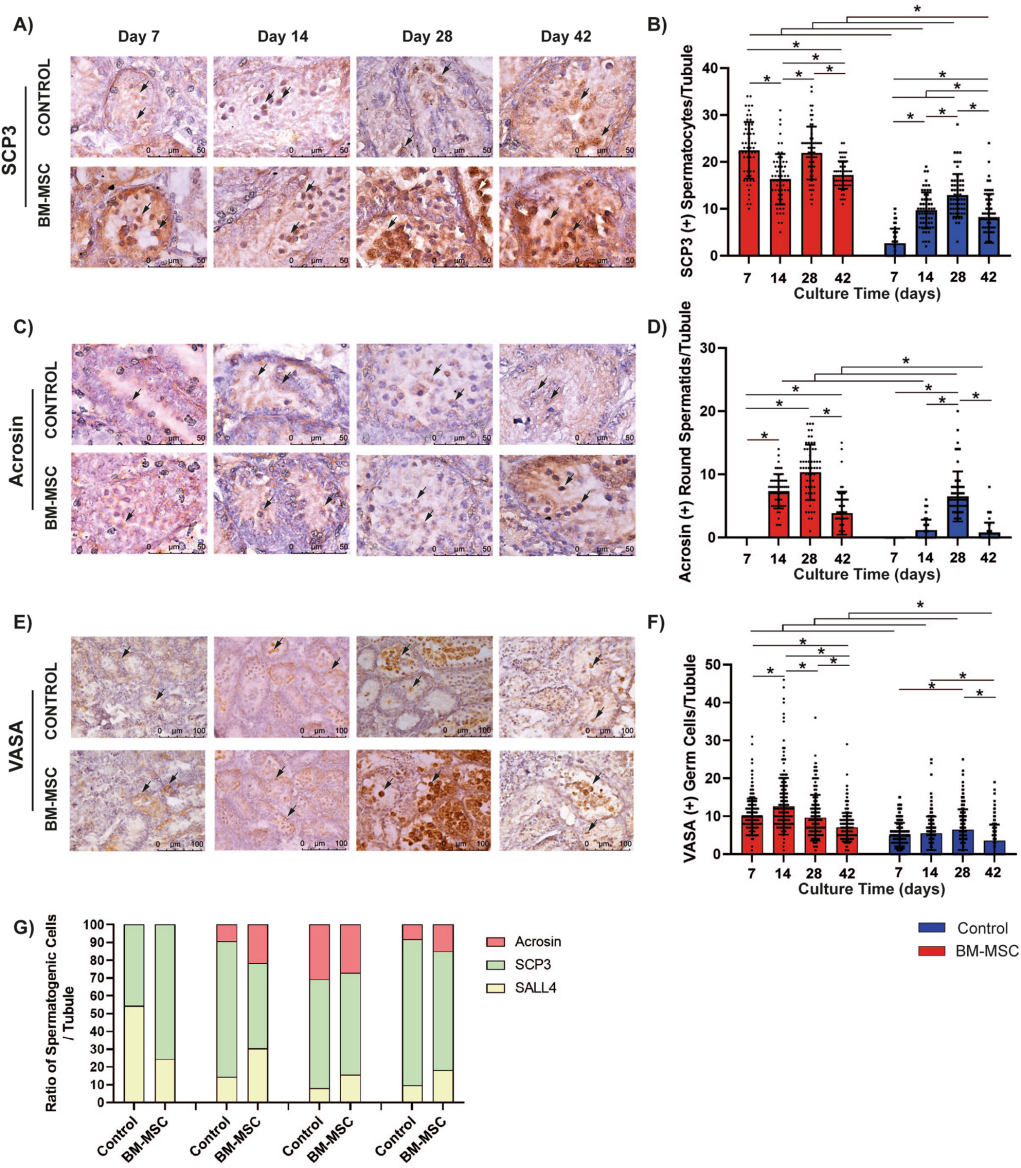
BM-MSCs promote spermatogenesis in vitro. Left column presents immune labeled micrographs of (**A**) SCP3, (**C**) Acrosin, (**E**) VASA in neonatal mouse testes cultured for 7, 14, 28 and 42 days (SCP3 and Acrosin: 1000x, VASA: 400x). Note the presence of spermatocytes and round spermatids indicated by arrow on SCP3 and Acrosin labeled sections, respectively. The right column presents bar graphs illustrating standard deviation and data distribution of time dependent change in (**B**) SCP3(+) (**D**) Acrosin(+) (**F**) VASA(+) GCs per seminiferous tubules (*p < 0.05, n = 6 testes, 300 tubules for VASA, 60 tubules for SCP3 and Acrosin). (**G**) The band graph represents the ratio of spermatogenic cells in seminiferous tubules (yellow: SALL4(+) SSPCs, green: SCP3(+) primary and secondary spermatocytes, red: Acrosin(+) spermatids).

The number of SCP3(+) primary and secondary spermatocytes per tubule on days 7, 14, 28 and 42 and Acrosin(+) round spermatids on days 14, 28 and 42 in the BM-MSC co-culture group were higher by IHC when compared to the control (*p = *0.001, Fig. 4A,B). The SCP3(+) spermatocytes increased (*p = *0.001) from day 14 to 28 and decreased (*p = *0.001) from day 7 to 14 and day 28 to 42 in the BM-MSC co-culture group (Fig. 4B, Supplementary Fig. S1D). The quantity of SCP3(+) spermatocytes in the control group increased with time from day 7 to 28 (*p = *0.001), but decreased however from day 28 to 42 (*p = *0.001, Fig. 4B). In total, the SCP3(+) spermatocyte number per tubule was higher on day 42 in the BM-MSC co-culture platform than on day 7 in the control group (*p = *0.001, Fig. 4B).

The BM-MSC co-culture group exhibited an increase in Acrosin(+) round spermatid numbers per tubule from day 7 to 14 (*p = *0.001) and day 14 to 28 (ns). A decline in Acrosin(+) round spermatids was apparent between days 28 to 42 (*p = *0.001, Fig. 4C,D, Supplementary Fig. S1E). Elongated Acrosin(+) spermatids were not detected by IHC on seminiferous tubules.

The quantity of VASA(+) GCs per tubule was significantly higher in the BM-MSC co-culture group on days 7, 14, 28 and 42 by IHC (*p = *0.001, Fig. 4E,F, Supplementary Fig. S1F). A significant increase of VASA(+) cell number was observed from day 7 to 42 (*p = *0.001, Fig. 4F) in the BM-MSC co-culture group while it exhibited an increase from day 7 to 28 (*p = *0.001) and a decrease from day 28 to 42 in the control (*p = *0.001).

Quantitative analysis of the ratio of SALL4(+) SSPCs, SCP3(+) spermatocytes and Acrosin(+) spermatids in testes contributing to the progression of spermatogenesis demonstrated that a significant advantage for the use of BM-MSCs when compared to the control (Fig. 4G, Supplementary Table 1).

### BM-MSCs support testicular maturation by increasing the tubular and luminal areas in vitro

**Figure 5:**
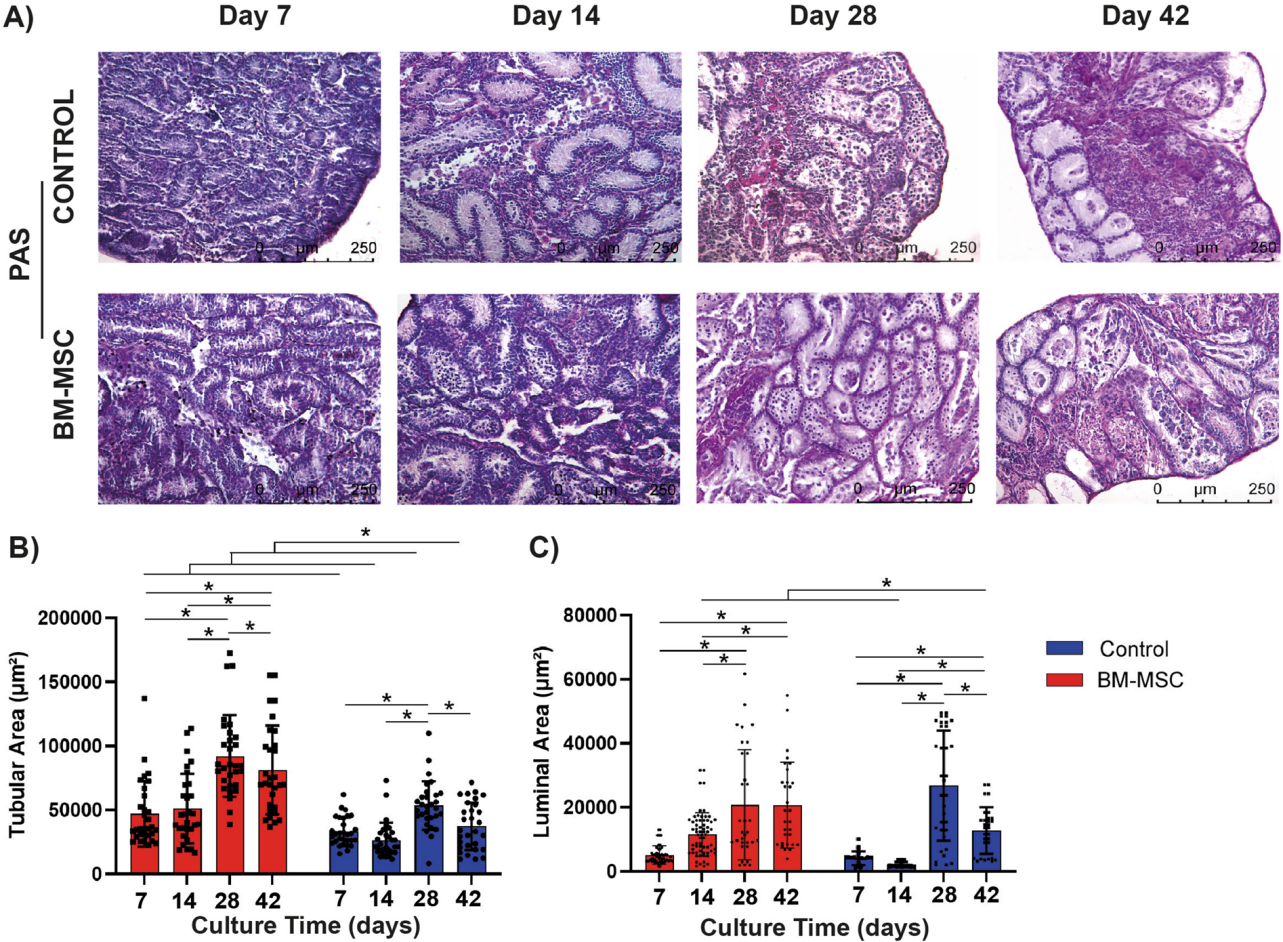
BM-MSCs promote testicular maturation in vitro. Micrographs presenting cultured mouse testes in (**A**) BM-MSC and control groups on days 7, 14, 28 and 42 (PAS-Hematoxylin, 100x). Bar graphs illustrate time dependent change by standard deviation and data distribution of (**B**) tubular and (**C**) luminal area (*p < 0.05, n = 6 testes, 300 tubules).

The BM-MSC co-culture group presented a larger seminiferous tubular area when compared to the control during the whole culture period (*p = *0.001, Fig. 5A,B). The BM-MSCs induced an increase of the tubular area from day 7 to 14 (ns) and 14 to 28 (*p = *0.001, Fig. 5B). A decline in the tubular area was observed from day 28 to 42 in this group (*p = *0.001). In the control group, the tubular area decreased from day 7 to 14 (ns) and 28 to 42 (*p = *0.001). This group revealed an increase in the tubular area between 14 and 28 days (*p = *0.001). The luminal area of the BM-MSC co-culture group was larger than that of the control on days 14 and 42 (*p = *0.001, Fig. 5A,C). They were similar to each other on days 7 and 28. The luminal area exhibited a continuous enlargement in the BM-MSC co-culture group and this was significant from day 14 to 28 (*p* = 0.001). The control group presented an increased luminal area from day 14 to 28 (*p* = 0.001) and a decline was found between day 7 to 14 (ns) and day 28 to 42 (*p = *0.001, Fig. 5C).

### BM-MSCs induced increased numbers of testicular cells in G2-M phase of the cell cycle and cellular viability in vitro

**Figure 6:**
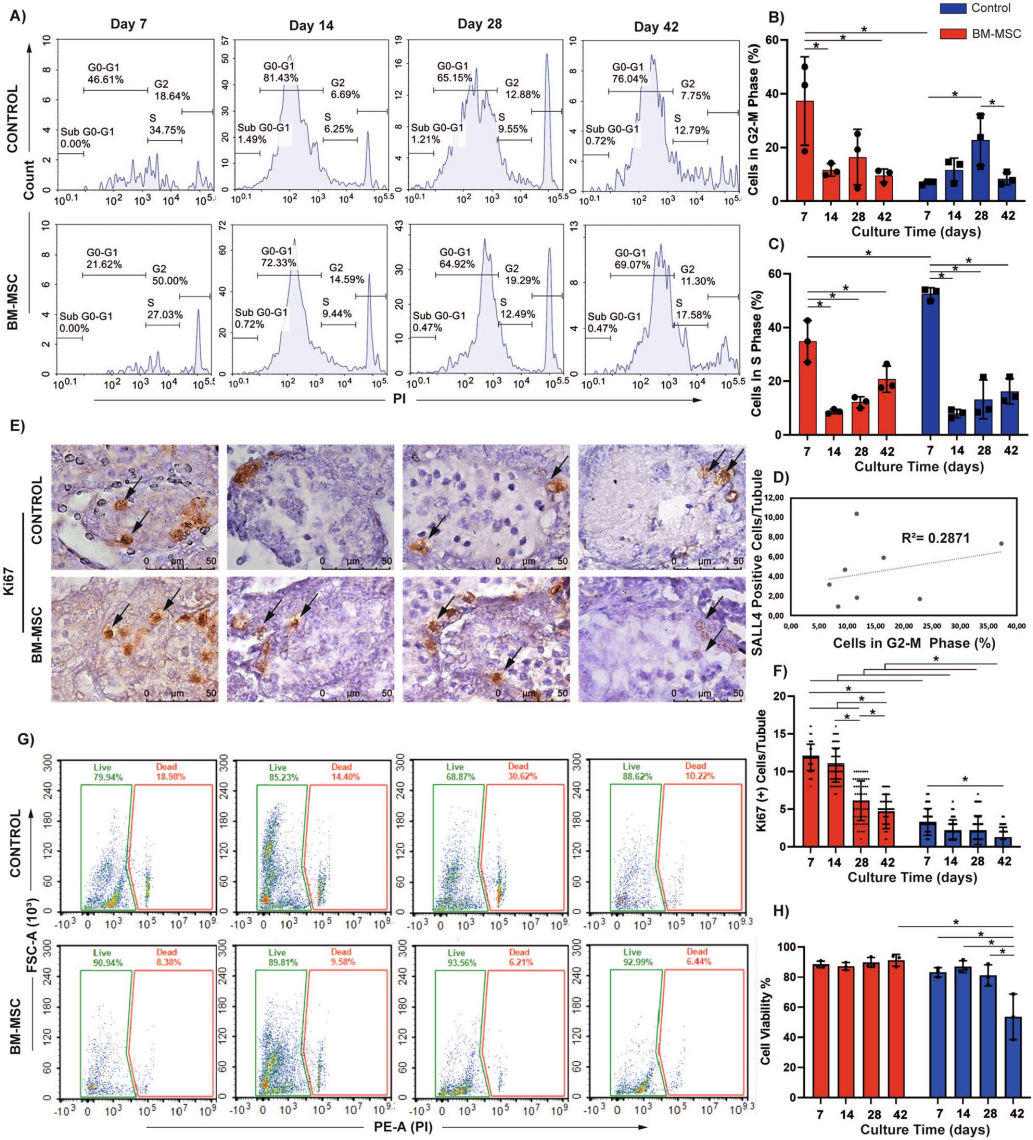
BM-MSCs stimulate mitotic phase of the cell cycle for 7 days and induce cell viability and proliferation for 42 days of in vitro testis organ culture. (**A**) Flow cytometric analysis of cell cycle is illustrated. The time dependent change in (**B**) G2-M and (**C**) S phases in BM-MSC co-culture and control groups is shown by bar graphs with standard deviation and data distribution (*p* < 0.05, n = 3). (**D**) Line graph illustrates the positive correlation between number of SALL4(+) SSPCs and percentage of cells in G2-M phase in control and BM-MSC groups (R^2^ = 0.2871, *p* < 0.05). (**E**) Ki67(+) testicular cells in neonatal mouse testes cultured for 7, 14, 28 and 42 days (1000x). (**F**) The time dependent change in number of Ki67(+) testicular cells in BM-MSC co-culture and control group is illustrated in bar graph with standard deviation and data distribution (*p < 0.05, n = 6 testes, 60 tubules). (**G**) Flow cytometric analysis of live/dead cell and (**H**) in time change in cell viability in testes are shown (*p < 0.05, n = 6 testes).

The percentage of total testicular cells that were in G2-M phase was higher in the BM-MSC co-culture group with respect to the control by FCM on day 7 (*p = *0.03, Fig. 6A,B). The numbers of cells in S-phase remained constant in both groups during the whole culture period (Fig. 6A,C). The SALL4(+) SSPC count in the BM-MSC and the control groups by IHC presented a positive correlation with testicular cell number in the G2-M phase by FCM (R^2^ = 0.2871, *p = *0.026, Fig. 6D).

BM-MSCs increased the number of Ki67(+) proliferative spermatogonia per tubule from day 7 to 42 when compared to the control by IHC (*p = *0.001, Fig. 6E,F, Supplementary Fig. S1G). The quantity of Ki67(+) cells remained constant from day 7 to 14 (ns), and demonstrated a decline from this point forward until day 42 in the BM-MSC co-culture group (*p = *0.001, Fig. 6F). The control group presented a continuously low number of Ki67(+) cells with a decline from day 7 to 42 (*p = *0.001).

BM-MSCs increased the number of viable cells in the testes (> 80%) on day 42 when compared to the control by FCM (*p = *0.001, Fig. 6G,H). The control group however presented a sharp decline in percentage of viable cells from day 14 to 42 (*p = *0.001, Fig. 6H).

## Discussion

This study revealed that syngeneic BM-MSCs support the maintenance of SSCs and expansion of SSPCs for 42 days, spermatogenic lineage differentiation by increasing the number of spermatocytes and spermatids for 28 days, mitosis of total testicular cells for 7 days, testicular cell proliferation and survival for 42 days in C57BL/6 neonatal mouse testes within an ALI co-culture platform in vitro. Here, we successfully designed and validated a sustainable testicular organ culture platform for expansion of the SSPC pool and induction of spermatogenesis in neonatal mice using an ALI system^22^ with a co-culture consisting of the addition of syngeneic BM-MSCs. Our group previously reported a limited capacity of neonatal C57BL/6 mice-SSPC proliferation in monolayer cultures with leptin^23^. Similarly, human SSPCs in monolayers present inadequate colonization capacity *in vitro*^24^. Testicular and gonadal cultures are assessed for their support of SSPC maintenance of humans^25,26^, mice^27–29^, rats^30^ and goats^31^ for extended periods in 3D ALI^28,29^, hanging drop^31^ and modified soft agar^32^ or alginate^27^ culture systems and compared to monolayers. We designed a syngeneic BM-MSC-supported indirect co-culture environment that provides a real-time cellular paracrine contribution to in vitro newborn mouse testicular maturation including complete spermatogenic cycle in this study. The BM-MSCs present an easy, reliable autologous and allogeneic cellular source for personalized therapies in the clinics including cancer^33^, metabolic diseases^34^ and also infertility^35^. Previous in vivo studies revealed a regenerative effect of bone marrow^14,15,36^, adipose tissue^13,37^ and umbilical cord^12,18,38^ derived MSC transplantation on chemically sterilized adult mouse^12,18^, rat^13,14,37,38^ and hamster^15,36^ testes in terms of paracrine immune modulation^39^, GC number^12,40^ and seminiferous tubular and luminal area^14^. The BM-MSCs which constitute the essential factor in our platform share common origin and paracrine secretory profile^9^ with Sertoli cells presenting supporting functions for self-renewal, maintenance of spermatogonia and preservation of spermatogenesis^11,41^. For this reason, we chose MSCs derived from bone marrow in our study to enhance the spermatogenetic potential in 3D ALI testis organ culture platform in vitro.

Here we report that expansion of SALL4 and OCT4(+) SSPCs (IHC) and maintenance of ID4(+) SSCs during 42 days of co-culture with syngeneic BM-MSCs was increased when compared to controls and SALL4 data was quantitatively correlated with PLZF labelling as measured by FCM. Stable nuclear co-localization of PLZF and SALL4 transcription factors secure the maintenance of the SSPC pool in postnatal and adult mice testes^6^. While PLZF prevents stem cells from differentiation, this activity is suppressed by increased expression of the proliferative SSPC marker SALL4, and results in differentiation of SSPCs in the direction of spermatogenic lineages^6^. BM-MSCs increased numbers of SALL4, OCT4 and PLZF positive SSPCs during the first 14 days compared to controls, while a numerical decrease of SALL4 and OCT4 immune reactive cells was noted in both groups from this point forward until day 42 in our study. The PLZF immune reactivity percentage remained almost constant in time for 42 days in the BM-MSC co-culture and the control groups. PLZF expression was reported to be higher on day 40 of culture by RT-PCR in newborn mice seminiferous tubules in a modified soft agar system when compared to monolayer^42^. A time-related change in PLZF expression during the culture period was not reported in that study. In both agarose and alginate embedded adult mouse testes, the number of PLZF immune reactive cells per tubule by IHC decreased in time adult mice from day 0 to 36 with a gradient of maximum immune reactivity in marginal/submarginal areas to a minimum number in central compartments of the testicular strips. No significant difference was observed between agarose and alginate embedded tubules^27^. The BM-MSC ALI system supported a significant increase in PLZF positive cells in single cell suspensions of whole testicular specimens by quantitative FCM on day 7 and 14 when compared to the control. This dual increase in PLZF and SALL4 labelling supports the expansion of the SSPC pool by syngeneic BM-MSCs for the first two weeks of co-culture in the ALI platform. From this time forward, the higher SALL4 immune reactivity in BM-MSC groups contributed to differentiation of SSPCs in the BM-MSC co-culture groups when compared to our controls.

Here we demonstrate that the number of SALL4(+) SSPCs per tubules were as high as 6 ± 12 and 1.55 ± 11 on day 28 with and without BM-MSCs, respectively, on the ALI platforms at day 42. Komeya et al. ^43^ established similar ALI platforms using newborn Acr-GFP transgenic mice testes and compared these with a PDMS-sealed ALI setup for 54 days of culture. They reported the number of Acr(-) (Acrosin is specific to round spermatids and spermatozoa) and GFRa1(+) SSCs per tubule as near to 1.0–1.5 and 0.5–1.0 on day 28 as a single time point in ALI and PC-ALI platforms, respectively. GFRa1 marker labels only the SSCs and spermatogonial progenitor cells are not included in this population^44^. In contrast, SALL4 is specific for both the stem and progenitor cell population^45^. In our study, BM-MSCs clearly increased the SSPC pool as evident from increased SALL4 labelling and was comparable to the number of SSCs by GFRa1 labelling to some extent. There is one report claiming that the 3D ALI co-culture of BM-MSCs (in concentration of 526 cells/cm^2^) with testicular cell suspension supports the differentiation capacity in GC direction, as evident from an increase in *Dazl* gene expression for 7 days in total in mice^46^. Our study evaluated the impact of syngeneic BM-MSC with 5435 cells/cm^2^ concentration on prepubertal testes in terms of testicular maturation, stem cell pool integrity/expansion and spermatogenesis. We replaced the BM-MSC feeder layer with fresh passage 3 cells every two weeks and we focused on the evaluation of changes in testicular cells instead of BM-MSCs. Previous in vivo studies reported the restoration of spermatogonia by counting number of GCs on H&E stained sections following intratesticular allogeneic BM-MSC injection with a single dose of 10^6^/total body weight in adult sterile rats and 1.75 × 10^5^/total body weight BM-MSCs in adult sterile hamster testes^14,47^. Our data on in vitro SSPC expansion is comparable with those of previous studies concerning restoration of spermatogonia in vivo in terms of the beneficial effect of the cells, but did not correlate with our in vitro newborn testicular culture setting.

In this study, we report that the syngeneic BM-MSCs induce IVS in an in vitro ALI platform in terms of increased numbers of VASA(+) total GCs^48,49^, SCP3(+) spermatocytes^50^ and Acrosin(+) spermatids^51^ for 28 days, the tubular and luminal area for 42 days, cell ratio in the S phase of the cell cycle for 7 days, cell proliferation and viability for 42 days by IHC, FCM and histology. We further evaluated whether the decrease in the SSPC pool after 14 days was due to undifferentiated spermatogonia undergoing spermatogenesis. We demonstrated the formation of spermatocytes and round spermatids from culture days 7 (13 dpp) and 14 (20 dpp) onwards, respectively in both the BM-MSC co-culture and the control groups. We chose these time points since during the first 7 days in the newborn mice, undifferentiated spermatogonia proliferate by mitosis and the SSPC pool expands. Meiosis begins from 7 dpp in mice and ends with the formation of spermatocytes on days 10–20, spermatids on days 18.8–25.3 and sperms on day 34.5^52^. The BM-MSCs contributed to SSPC expansion until day 14. From this time forward, numbers of SSPCs decreased simultaneously, differentiating into spermatogenic cells (spermatocytes, round spermatids and the total GC number per tubule) and increased in parallel with gradually expanding tubular and luminal areas in this group when compared to the control at day 42. Sato et al. revealed continued spermatogenesis up to round spermatids for 82 days by Acr-GFP expression in adult mice ALI testicular culture^53^. In our study, we maintained the GC population for 42 days by using a similar ALI culture platform with or without BM-MSCs, but the GC pool only increased in presence BM-MSCs. Recently, culture supplementation by AlbuMAX (10–40 mg/ml), hormones (Testosterone, 3,3′,5-Triiodo-L-thyronine sodium, LH, FSH), antioxidants (L-Ascorbic acid 2 glucoside, DL-α-Tocopherol acetate and L-Glutathione reduced) and lysophospholipids (L-α-Lysophosphatidylcholine and Lysophosphatidyl-serine) under low oxygen tension supported the development of round spermatids on day 42 of 70 days of culture using an ALI platform^30^. Our normoxic culture medium consisted of similar amount of KSR, without additional hormone, antioxidant or lysophospholipid supplementation. Our culture conditions supported the differentiation of a similar GC population to round spermatids from day 14 of neonatal mice ALI cultures, that was improved by co-culture with BM-MSCs. Round spermatids were noted after the first spermatogenic cycle in both studies when the length of rat and mice cycles were considered. Sato et al. were able to obtain functional spermatozoa from day 27 newborn mice with an ALI platform using 10% KSR, B-27, hepatocyte growth factor, Activin A, FSH, testosterone, BMP-4, BMP-7 and bovine pituitary extract^29^. Our culture medium lacks those additional supplements other than 10% KSR. However, the system contains BM-MSC-secreted soluble factors in the culture medium. Although the secretory profile of BM-MSCs was not assessed in this study, we postulate that BM-MSCs were not sufficient to induce terminal differentiation of round spermatids, as obtained by Sat’s supplementation. These results suggest that an IVS system based on syngeneic BM-MSC could improve not only the increase and preservation of the SSPC pool for at least 42 days, but also spermatogenesis up to the round spermatid stage in vitro from day 14. Our culture medium however needs to be enriched with additional supplements required to attain terminally differentiated, functional spermatozoon, and although the absence of full differentiation could be considered a minor limitation it does not affect the accuracy of the study.

Previous in vivo studies reported the therapeutic capacity and trans-differentiation potential of adipose-derived mesenchymal stem cells (AD-MSC)^13^ and BM-MSCs^14^ in terms of VASA/GFP(+) and SCP1/GFP(+) cells by IF in Wistar rat 12 weeks after intratesticular allogeneic GFP(+) AD-MSC injection with a single dose of 10^6 ^cells and BrdU labelled spermatogonia, spermatocyte, round spermatid and spermatozoa formation were confirmed by IHC 8 weeks after intratesticular injection of a single dose of 1.75 × 10^5 ^allogeneic BrdU(+) BM-MSCs injection with a single dose of 1.75 × 10^5^ in adult sterile rats. In our study, we generated an indirect communication between BM-MSCs and testicular cells and used their supportive potential to increase SSPC survival and spermatogenesis in vitro. Monsefi et al. reported that the intratesticular injection of 10^6^ allogeneic BM-MSC/ injection in 10^6^/total body weight concentration increased the tubular and luminal area in sterile rat testes after 60 days of injection^14^. Similarly, we demonstrated in our organ culture setup an increase in the tubular and luminal area using the ALI platform in combination with BM-MSCs for 42 days in mice. Yang, R F et al. reported that the in vivo intratesticular injection of 10^5^ MSCs into azoospermic mice leads to the expansion of spermatogonia numbers and genes consistent with *germline specific markers (miwi*, VASA) and spermatocyte marker (SCP3) at 3 weeks after injection by Western Blotting^12^. In line with this study, we demonstrated the capacity of BM-MSCs to increase numbers of VASA immune reactive GCs, SCP3(+) spermatocytes and SALL4/OCT4/ID4(+) spermatogonia by IHC for after a 42-day long in vitro culture period by IHC. Tamadon et al. demonstrated that a single intratesticular injection of 10^6^ allogeneic BM-MSC into sterilized hamsters induced spermatogenesis up to spermatozoa and increased tubular diameter on day 60 when compared to controls by H&E staining^15^. The change in tubular diameter in Tamadon’s study is consistent with our study demonstrating an increase in tubular area after culture with BM-MSCs for 42 days within an in vitro ALI platform. However, these in vitro conditions do not reflect the physiological in vivo microenvironment. In addition, animal models consist of chemically induced sterilization models in adult animals. In our newborn mouse model, the spermatogenesis has not been initiated yet. The above mentioned in vivo experimental models thus may never reflect the in vitro maturation stages that are targeted within the scope of our ALI model. The possible advantages of BM-MSCs in in vitro and in vivo experimental settings in terms of being a possible candidate for personalized cellular therapeutic agents could be comparable to some extent.

The modification in the ALI system using a BM-MSC indirect co-culture system increased the SSPC pool, immature testis maturation and induced the spermatogenesis until round spermatids in vitro. This study lacks the molecular characterization of SSCs by qPCR at the mRNA level. However, we characterized the SSCs at the protein level by immune labelling against the ID4 antigen, which is specific for single spermatogonia (self-renewing SSCs)^4^. The final stage of spermatogenic cells obtained from culture is round spermatids. Elongated spermatids were not obtained in culture with/without BM-MSCs. Our ALI platform did not provide complete spermatogenesis by formation of elongated spermatids, which may be due to the absence of critical supplements, required for the spermatogenic cycle, such as testosterone, bFGF, GDNF or LIF^29,54^. Nevertheless, obtaining round spermatids is an advantage for getting offspring since ROSI is a commonly used method included in assisted reproductive technology in the clinics. Currently, ROSI is a crucial clinical intervention to allow fertilization using cells from males, with infertility from different etiology. The round spermatids obtained using this setup need to be tested for their functionality and safety with ROSI and mutation analysis with further in vitro*/*in vivo animal experiments. Nevertheless, these results exhibit a great example of the supportive effect of BM-MSCs in the generation of an expanded pool of round spermatids in vitro. Our platform used to imitate the prepubertal period has, however, several limitations. Our results exhibit an ex vivo culture platform that needs to be improved via further in vivo studies evaluating the functionality of GCs in engraftment and fertilization after transplantation. On the other hand, our findings are reliable since the statistical relevance and parametric distribution of the samples were confirmed. Our in vitro results should be taken one step forward by transplantation of testicular strips which are cultured with BM-MSCs into infertile mice in order to reveal the functionality of round spermatids obtained from culture. Although enumeration of Sertoli cells with immunolabeling was not performed, we did show that change in testicular morphology was associated with Sertoli cell maturation and viability. These data are valid and reliable to gain information about Sertoli cell maturation and number^55,56^. The decrease in total GC numbers from day 14 might be explained by considering the static culture setups used in our study. When compared to static platforms consisting of an ALI, microfluidic devices may be even capable of maintaining spermatogenesis for longer time periods^57–59^. In addition, the secretome profile of syngeneic BM-MSCs and the genetic stability of obtained round spermatids should be assessed in further studies.

In conclusion, the overall outputs revealed that syngeneic BM-MSCs can promote survival, expansion and differentiation of the SSPC pool and maturation of prepubertal testes in vitro for 42 days. The cryopreserved testis strips carrying increased numbers of SSPC and spermatogenic cells can be stored until the cytotoxic cancer treatment finishes and the SSPCs/spermatogenic cells isolated from the testis strips can be transplanted back to the testicular tubules when the individual reaches adulthood. Obviously, this whole procedure needs to be validated in humans. In conclusion, addition of BM-MSCs to the ALI co-culture system provide a promising tool for a personalized cellular therapy platform to further develop in vivo and clinical studies for preservation of fertility in childhood cancer survivors focusing on expansion of the SSPC pool and initiation of spermatogenesis in immature testis strips in vitro.

## Methods

The study was designed and carried out in compliance with the ARRIVE guideline. We designed a prospective, randomized, controlled, double blinded (outcome assessment and data analysis) study. Independent variables are time (7, 14, 28 and 42 days) and groups (control and BM-MSC co-culture), dependent variables are markers of SSPC pool (SALL4 and PLZF), differentiating GCs (c-Kit) and total GCs (VASA); number of spermatogonia, spermatocytes and round spermatids; percentage of phases in cell cycle; and tubular-luminal area. Six animals for each group (n = 6, n = 48 in total) were used according to the result of power test, performed by G-power 3.1.9.7 with medium effect size (0.3) and 5% margin of error, in order to determine the appropriate sample size for this study at the beginning. Power analysis was performed with the "t-test on dependent groups" method. Therefore, the number of animals was determined as n = 48 (96 testes) for an equal number of testes (6 testes per group) to each of the experimental and control groups at four observation points on days 7, 14, 28 and 42, where 2 sets of experiments were to be carried out for histological and flow cytometric analyses, separately. In order to obtain the necessary number of BM-MSCs, n = 5 newborn mice were used (Fig. 1).

### Animals

Six-day old C57BL/6 male mice were purchased from the Experimental Animal Breeding and Application Center (Başkent University, Ankara, Turkey) and were used as organ and cell source. Mice were kept in air-conditioned, pathogen free rooms with 12 h light and 12 h dark lightning cycle at temperature of approximately 20 ± 2 °C and humidity of 55 ± 5%. The mice were fed with standard mouse diet and received tap water ad libitum. The methods used in all animal experiments conformed to the Guide for the Care and Use of Laboratory Animals and were approved by The Local Ethical Board of Animal Experiments (Hacettepe University, Ankara, Turkey (#52338575–96)).

### Isolation and culture of BM-MSCs

Six days old neonatal mice (n = 5) were used as a BM-MSC source. Briefly, mice were sacrificed by cervical dislocation and sterilized with 70% ethanol. Incisions were made at the beginnings of hind limb, and tibia/femur bones were removed. The samples were placed into phosphate buffer saline (PBS) solution (pH = 7.4, Thermo Fisher Scientific, USA) including 1% Penicillin Streptomycin Solution (pen-strep) (Biological Industries, Israel). After removal of the surrounding tissue, the bone samples were washed by PBS including 1% pen-strep and put into culture medium prepared with α-Minimum Essential Medium (α-MEM) (Biological Industries, USA) supplemented with 15% Fetal Bovine Serum (FBS) (Biological Industries, USA) and 1% pen-strep. The bones were washed with culture medium through 5 ml syringe from ends of cavities. The obtained cell suspension was cultured with 5% CO_2_ at 37 °C^60^.

### Characterization of BM-MSCs

All characterization studies were processed with MSCs at passage 3 which are used in whole co-culture experiments as recommended previously^15,61–63^.

*Morphologic Evaluation:* The obtained passage 3 BM-MSCs were evaluated according to their morphology and adhesiveness capacity to the culture flask.

*Surface Marker Analysis by FCM:* Passage 3 BM-MSCs were distributed as 2 × 10^5^ cell/tube within Hank’s Balanced Salt Solution (HBBS) (pH = 7.15–7.5, Biological Industries, Israel). The cells were labeled with CD44, CD140a, Sca-1 (BD Biosciences, USA) antibodies which are mouse MSC surface markers; and CD34, CD45 (BD Biosciences, USA) mouse hematopoietic cell surface markers^64^. PE- Rat IgG2b, APC-Rat IgG2a, PE-Rat IgG2a, PE-Rat IgG2a and FITC- Rat IgG2b (BD Biosciences, USA) were used respectively as isotype control antibodies. Measurements were performed with a Novocyte (ACEA Biosciences, USA) flow cytometer and results were analyzed using Novoexpress 1.3.0. (ACEA Biosciences, USA) software with 10,000 events recorded for each sample.

*Adipogenic Differentiation:* When BM-MSCs reached 100% confluence; the culture medium was changed with adipogenic differentiation medium prepared by addition of 10^–8^ M dexamethasone, 10 μg/ml insulin, 50 μM indomethacin and 0.5 μM 3-isobutyl-1-methylxantine (IBMX) in α-MEM medium^60^. After the initiation of differentiation, the cells were cultured through 21 days. Oil Red O (ORO) staining was performed in order to make morphological examination of differentiation. For semi-quantitative analysis, ORO in cells was extracted and measured via spectrophotometric micro plate reader (Tecan, Australia) at 492 nm wavelength.

*Osteogenic Differentiation:* When BM-MSCs became 70% confluent, BM-MSC culture medium was changed with osteogenic induction medium prepared by addition of 10 nM dexamethasone, 50 mM L-ascorbic acid and 20 mM β-Glycerophosphate to α-MEM medium^65^. After the initiation of osteogenic differentiation, the cells were cultured through 21 days. Alizarin Red S (ARS) (pH = 4.2, Sigma, USA) staining was performed in order to make morphological examination of differentiation. For semi-quantitative analysis, the amount of calcium in cells were measured by Quantichrom™ Calcium Assay kit (Bioassay Systems, USA) via spectrophotometric microplate reader (Tecan, Australia) at 492 nm wavelength.

### Testicular tissue collection and preparation

Six days neonatal mice (n = 48) were euthanized by cervical dislocation before the removal of testicular tissue. The abdominal skin was cut with surgical scissors and opened until the testicules. Then, the testes were removed by using forceps and placed into 4 °C HBSS supplemented by 1% pen-strep. Left (control group) and right (experiment group) testes of each animal were put into separate petri dishes and decapsulated under stereomicroscope (Leica, Weitzlar, Germany). Each testis was divided into 3 pieces and placed onto agarose gel in a dish in order to get approximately 1 mm^3^ sized strips which is necessary to keep the diffusion sufficient during the culture period (Fig. 1)^29^. The testis strip carrying agar pieces were placed in ALI culture systems and maintained for days 7, 14, 28 and 42 (n = 12, each).

### Establishment of BM-MSC Testis co-culture

BM-MSCs were seeded as 50,000 cell/well 4 days before the experiment in order to achieve 70% confluence to get a sufficient paracrine signaling during the culture period. Cells were grown with 15% FBS and 1% pen-strep supplemented α-MEM medium at 5% CO_2_ at 37 °C. Co-cultures were put onto transwell inserts with a 0.4 µm pore size (C3450, Corning, USA). Testes were cultured in 10% Knockout™ Serum Replacement (KSR), 1% pen-strep supplemented α-MEM medium at 5% CO_2_ at 34 °C. Culture medium was changed every other day (Fig. 1).

### Histochemistry and histomorphometry

Testicular strips were fixed in Bouin’s fixative, processed by automated vacuum tissue processor (Leica, Germany) and embedded in paraffin by using an embedding station (Leica, Germany). Three µm thick paraffin sections were stained by Periodic Acid Schiff Kit (PAS) (Sigma, USA) and evaluated under digital camera attached light microscope (Leica DMR 6000, Germany). Tubular and luminal areas^66–69^ of fifty seminiferous tubule sections per sample were measured by using an image analysis program (LASv3 Leica, Germany) and recorded.

### Flow cytometry

Testicular single cell suspensions were prepared for FCM analysis according to the literature^70^. Briefly, testes that completed the culture time were resuspended in 1 ml HBSS and the seminiferous tubules were disrupted mechanically by using syringe needle in a petri dish. The single cell suspension was then obtained by chemical digestion in an enzyme mixture of DNAse I (Serva, Israel) and 0.25% EDTA-Trypsin solution (1:9, v/v) for 30 min at 37 °C, and filtration through a cell strainer with a 40 μm pore size (Corning, USA). After washing with HBSS by centrifuging at 300*xg* at 4 °C, cells were labeled via APC-c-Kit (CD117), PE-PLZF and PI antibodies (BD Biosciences, USA). APC-Rat IgG2b and PE-Rat IgG2a were used as isotype control antibodies. Before the application of PI antibody, the cells were treated with RNAse. The measurement was performed by Novocyte (ACEA, Biosciences, USA) flow cytometer by reading 10,000 event for each. Novoexpress 1.3.0. (ACEA Biosciences, USA) program was used during analyses.

### Immunohistochemistry

Indirect immune peroxidase method^71^ was performed by using the Rabbit Specific HRP/DAB IHC Detection Kit (ab236466, Abcam, UK). Briefly, deparaffinized and rehydrated sections were incubated in H_2_O_2_ for 10 min at RT in order to block the endogenous peroxidase activity. Heat-induced antigen retrieval was carried out in citrate buffer (pH = 6.5). Protein blockage was performed by goat serum (Abcam, USA) incubation for 10 min at RT. The sections were incubated with anti-SALL4 (ab29112, Abcam, UK), anti-OCT4 (ab19857, Abcam, UK), anti-SCP3 (ab15093, Abcam, UK), anti-VASA (DDX4-MVH) (ab13840, Abcam, UK) and anti-Ki67 (ab15580, Abcam, UK) primary antibodies at 1:200 dilution for 25 min at RT, anti-ID4 (PA5-26,976, Invitrogen, USA) and anti-Acrosin (ab203289, Abcam, UK) primary antibodies at 1:200 dilution overnight at 4 °C. After primary antibodies, the sections were incubated with HRP-goat anti-rabbit IgG (Abcam, USA) secondary antibody for 15 min at RT. Then, DAB chromogen was applied to the slides for 5–7 min. The primary antibody incubation was omitted on negative control slides. Counterstaining was obtained by hematoxylin. Immune labeled cells were quantified in 300 seminiferous tubules of SALL4 and VASA, and 60 seminiferous tubules of OCT4, ID4, Ki67, SCP3 and Acrosin labeled sections of each group under brightfield microscope (Leica DM 6000 BM, Germany) attached camera by using image analysis program (LASv3 Leica, Germany).

### Statistical analysis

All experiments were performed as 2 paralleled and 3 repeats. Dependent variables were test parameters consisting of histomorphometry (tubular and luminal area), IHC (number of ID4, OCT4, SALL4, SCP3, Acrosin, Ki67 and DDX-MVH/VASA immune labelled cells); and FCM (the percentage of PLZF, c-Kit, PI(+) cells); the independent variables were experimental and control groups. The Shapiro–Wilk normality test evaluated the normality of distribution. The multiple comparison was performed by paired t-test and Wilcoxon signed-rank test. The parametric data were presented as mean ± S.E.M; the non-parametric data as median, minimum and maximum values. Analyses were performed at 95% confidence interval and margin of error of 5% on SPSS 16.0 Bivariate Correlation A program.

## Supplementary Information

Supplementary Information.

## Data Availability

All data generated or analyzed during this study are included in this published article (and its Supplementary Information file).
