# Supplementary material for: Mesenchymal stem cells promote spermatogonial stem/progenitor cell pool and spermatogenesis in neonatal mice in vitro
Source: Sci Rep. 2022 Jul 7;12:11494. doi: 10.1038/s41598-022-15358-5 (PMC9263145; doi:10.1038/s41598-022-15358-5)
Supplement: Supplementary file 1 — Supplementary Information. [file 41598_2022_15358_MOESM1_ESM.pdf]

SUPPLEMENTARY INFORMATION

Mesenchymal Stem Cells Promote Spermatogonial Stem/Progenitor Cell Pool and Spermatogenesis in Neonatal Mice *in vitro*

Selin Önen[1,2], Sevil Köse[3], Nilgün Yersal[4], *Petek Korkusuz[5]

1) Department of Stem Cell Sciences, Graduate School of Health Sciences, Hacettepe University, 06100, Ankara, Turkey

2) Department of Medical Biology, Faculty of Medicine, Atilim University, 06830, Ankara, Turkey

3) Department of Nutrition and Dietetics, Faculty of Health Sciences, Atilim University, 06830, Ankara, Turkey

4) Department of Histology and Embryology, Faculty of Medicine, Gaziosmanpaşa University, 60030, Tokat, Turkey

5) Department of Histology and Embryology, Faculty of Medicine, Hacettepe University, 06100, Ankara, Turkey

Supplementary Table 1. The ratio of SSPCs, spermatocytes and spermatids per tubule for each time point is given in the table.

	Culture Time (Days)	SALL4(+) SSPCs (%)	SCP3(+) Spermatocytes (%)	Acrosin(+) Spermatids (%)
**Control**	7	54.42	45.58	0.00
	14	14.57	76.23	9.20
	28	8.13	61.06	30.81
	42	9.84	82.07	8.09
**BM-MSC Co-culture**	7	24.62	73.38	0.00
	14	30.46	47.99	21.55
	28	15.52	57.40	27.08
	42	18.23	66.83	14.94

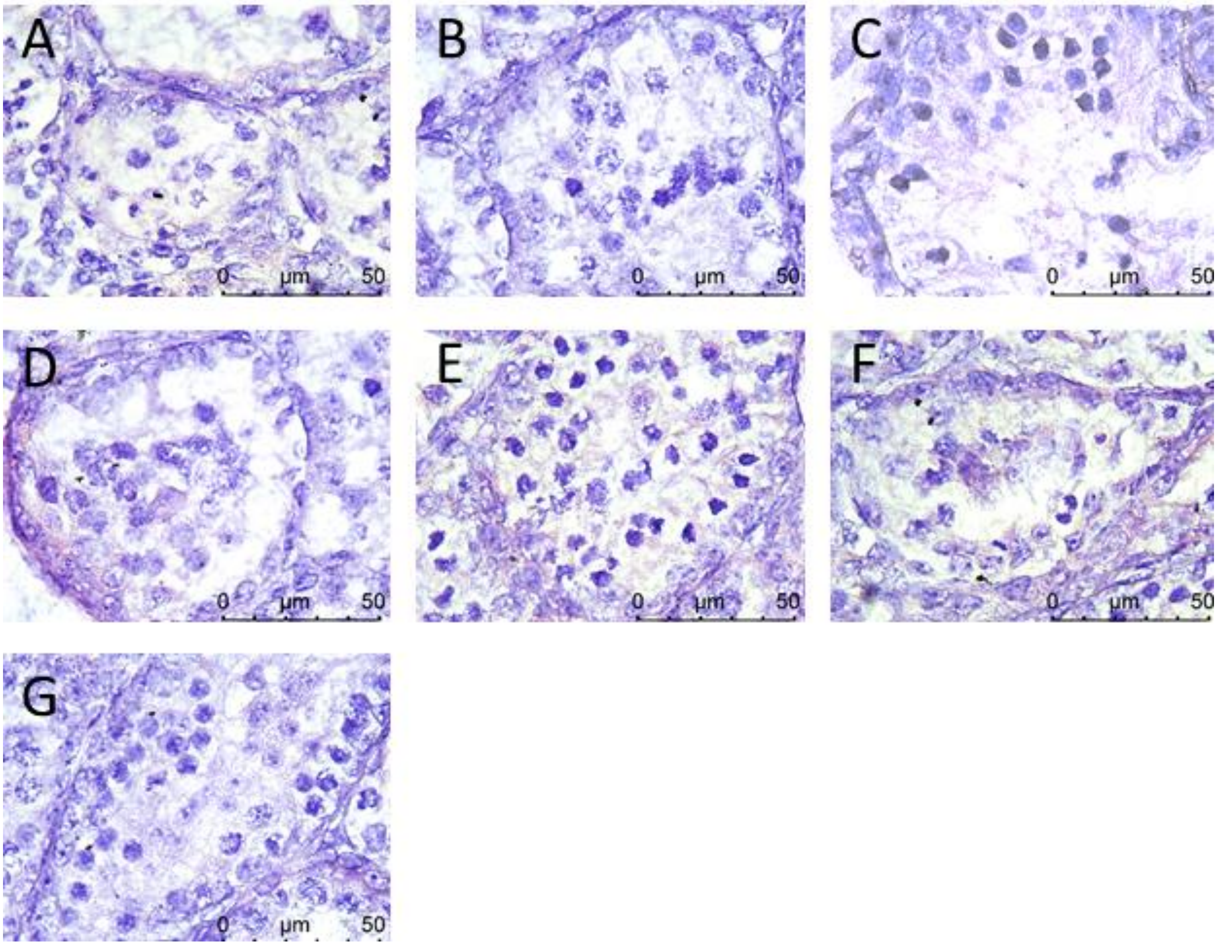

Supplementary Figure S1: Negative control micrographs presenting non-immunolabelling for the germ cell and proliferative cell specific markers: (A) anti-ID4, (B) anti-SALL4, (C) anti-OCT4, (D) anti-SCP3, (E) anti-Acrosin, (F) anti-VASA and (G) anti-Ki67 (1000x).
